# *In Vitro* Inhibitory and Cytotoxic Activity of MFM 501, a Novel Codonopsinine Derivative, against Methicillin-Resistant *Staphylococcus aureus* Clinical Isolates

**DOI:** 10.1155/2015/823829

**Published:** 2015-02-01

**Authors:** Saiful Azmi Johari, Mastura Mohtar, Sharifah Aminah Syed Mohammad, Rohana Sahdan, Zurina Shaameri, Ahmad Sazali Hamzah, Mohd Fazli Mohammat

**Affiliations:** ^1^Antimicrobial Laboratory, Anti-Infective Branch, Bioactivity Programme, Natural Products Division, Forest Research Institute Malaysia (FRIM), 52109 Kepong, Selangor, Malaysia; ^2^Faculty of Applied Sciences, Universiti Teknologi MARA (UiTM), 40450 Shah Alam, Selangor, Malaysia; ^3^Biotherapeutic Branch, Bioactivity Programme, Natural Products Division, Forest Research Institute Malaysia (FRIM), 52109 Kepong, Selangor, Malaysia; ^4^Organic Synthesis Laboratory, Institute of Science, Universiti Teknologi MARA (UiTM), 40450 Shah Alam, Selangor, Malaysia

## Abstract

28 new pyrrolidine types of compounds as analogues for natural polyhydroxy alkaloids of codonopsinine were evaluated for their anti-MRSA activity using MIC and MBC value determination assay against a panel of *S. aureus* isolates. One pyrrolidine compound, MFM 501, exhibited good inhibitory activity with MIC value of 15.6 to 31.3 *μ*g/mL against 55 *S. aureus* isolates (43 MRSA and 12 MSSA isolates). The active compound also displayed MBC values between 250 and 500 *μ*g/mL against 58 *S. aureus* isolates (45 MRSA and 13 MSSA isolates) implying that MFM 501 has a bacteriostatic rather than bactericidal effect against both MRSA and MSSA isolates. In addition, MFM 501 showed no apparent cytotoxicity activity towards three normal cell lines (WRL-68, Vero, and 3T3) with IC_50_ values of >625 *µ*g/mL. Selectivity index (SI) of MFM 501 gave a value of >10 suggesting that MFM 501 is significant and suitable for further *in vivo* investigations. These results suggested that synthetically derived intermediate compounds based on natural products may play an important role in the discovery of new anti-infective agents against MRSA.

## 1. Introduction

Methicillin-resistant* Staphylococcus aureus* (MRSA) is a nosocomial-related, Gram-positive bacterium that has been known to display multidrug-resistance properties towards a wide range of structurally unrelated antibiotics and antimicrobial agents. Currently, only a handful of antibiotics such as vancomycin and linezolid could inhibit this dangerous pathogen. Economically, the estimated cost of antibacterial usage in the Ministry of Health (MOH) hospitals in Malaysia has risen from RM 90 million/year in 2006 to RM 112 million in 2008 while MRSA is still the second most isolated pathogen from blood in MOH hospitals [1–3]. Recently, heterovancomycin-resistant* S. aureus* (hVRSA) has been detected in Malaysia which suggests that local MRSA strains have started to develop reduced susceptibility properties against vancomycin [4, 5]. Additionally, a local study has also detected the relatively high presence of community-acquired (CA) MRSA in healthy university students [6]. Another related study also shows that there are 3% of CA-MRSA isolates among hospital-associated MRSA isolates from four MOH hospitals [7]. Presence of CA-MRSA is considered quite serious since these isolates not only are limited in healthcare settings, but also have spread to healthy individuals that are not related to hospitals [8]. These reports indicate that we are not safe from this emerging infectious disease and the treatment of MRSA will cost our country and other developing countries economical loss of more millions if not billions of dollars as compared to developed nations [9, 10]. A dire need for a new and alternative source of antimicrobials is very crucial to prevent or at least reduce MRSA infections worldwide.

Natural polyhydroxy pyrrolidine alkaloids are known to display remarkable biological properties such as potent antibiotic, antiviral, and antifungal properties [11]. However, pyrrolidine alkaloids carrying an aromatic substituent on the ring are of a rare class found in nature [12]. Codonopsinine and codonopsine isolated from* Codonopsis clematidea* are two examples in this unusual category [13, 14]. The aerial parts of this perennial herb, commonly known as Asian bellflower, have been reported to contain both of these compounds which exhibited antibiotic and hypotensive activities and low toxicity level and do not affect the central nervous system [15]. Traditionally, the aerial parts of this plant have been used as a cholagogue to treat liver disease and hepatitis and jaundice and were used in combination with other folk medicines to improve hepatic functions in Uzbekistan [16, 17]. Total synthesis of the codonopsinine compound has been a challenge to many organic synthetic chemists due to its structural complexity although several approaches have successfully produced the desired compound [18–21]. Previously, we have synthesized several derivatives of codonopsinine representing interesting electron rich functional group identified to be responsible for many pharmacological properties using a one-pot reaction [22, 23].

In this study, 28 new codonopsinine derivatives were prepared and evaluated for their anti-MRSA activity and safety profile using minimum inhibitory concentration (MIC) assay, minimum bactericidal concentration (MBC) assay, and* in vitro* cytotoxicity assay.

## 2. Material and Methods

### 2.1. Preparation of Codonopsinine Derivatives

All 28 codonopsinine derivatives were synthesized using a one-pot reaction and confirmed using NMR and FTIR methods. Briefly, diethyl oxalate (1 equivalent), amine (1 equivalent), and aromatic aldehyde (1 equivalent) will be mixed in ethanol solution. The solution will then be heated and stirred under reflux for 1 h. After cooling, the mixture will be added on ice water and then acidified with HCl. The precipitate will be filtered and washed with water and diethyl ether to remove traces of aldehyde to yield the pure cyclized product. The exampled procedures for preparation, characterization, and identification of several codonopsinine derivatives used in this study were as described previously [22, 23].

### 2.2. Bacterial Isolates and Growth Conditions

Forty-three MRSA and 11 MSSA clinical isolates from three local hospitals with additional three American Type Culture Collection (ATCC) MRSA reference strains (ATCC 33591, ATCC 700699, and BAA-1556) and two MSSA ATCC reference strains (ATCC 25923 and ATCC 35556) were used in this experiment (as listed in Table 1). All of these strains have been identified and characterized previously [24, 25] except for isolates HS770, HS3175, and HS3178 and isolates A1 to D5 were first used in this communication. Isolates were maintained on Protect Bacterial Preservers (Technical Service Consultants Limited, Heywood, Lancashire, England) at −20°C. Prior to use, isolates were subcultured overnight at 37°C in Mueller-Hinton broth (MHB), adjusted to obtain a turbidity comparable to that of 0.5 McFarland standard. Isolates N441, U949, ATCC 25923, and ATCC 33591 were chosen as a preliminary panel for MIC evaluation due to their active efflux properties and different susceptibility profiles against five commonly known efflux substrates, namely, erythromycin, norfloxacin, tetracycline, chloramphenicol, and ethidium bromide as published previously [24, 26, 27]. Hence, the preliminary isolates are not only multidrug-resistance towards various types of antibiotics [24] but with active efflux properties as well [26], making them a formidable panel of MRSA/MSSA isolates to be tested against any potential anti-MRSA agents.

### 2.3. Determination of Minimum Inhibitory Concentration (MIC)

Initial MIC value determination assay was carried out to evaluate the 28 derivatives potential as inhibitory agent against a panel of test isolates (N441, U949, ATCC 33591, and ATCC 25923) using a double-broth microdilution method involving 96-well microtiter plates as described previously [27]. Briefly, serial twofold dilutions of the test compounds dissolved in dimethyl sulfoxide (DMSO) were prepared prior to addition of 100 *μ*L overnight microbial suspension (10^8^ cfu/mL) followed by incubation at 37°C for 24 hrs. The highest concentration of DMSO remaining after dilution (5%, v/v) caused no inhibition of bacterial growth. The MIC value was defined as the lowest concentration producing no visible growth (absence of turbidity and/or precipitation) as observed through naked eye. For further reconfirmation, 20 *μ*L (1 mg/mL) of 3-(4,5-dimethylthiazol-2-yl)-2,5-diphenyltetrazolium bromide (MTT) reagent was added to the bacterial suspension in the selected wells, followed by 20 minutes of incubation at 37°C. The reagent-bacterial suspension colour will remain clear/yellowish for inhibitory activity as opposed to dark blue indicating growth. Following that, compounds that showed good inhibitory activity (MIC < 64 *μ*g/mL) will be evaluated further using additional MRSA/*S. aureus* isolates. Standard norfloxacin antibiotic was also used in this study for comparison.

### 2.4. Determination of Minimum Bactericidal Concentration (MBC) and MBC/MIC Ratio Values

The MBC values were obtained by subculturing the contents of each negative well and from the positive control (broth with inoculum, without compound) of MIC determination, onto substance-free Mueller-Hinton agar (MHA) Petri dishes. The plates were incubated at 37°C for 24 hrs. The MBC was taken as the lowest concentration of substance that results in more than 99.9% reduction of the initial inoculum. Results were expressed as mean values of three independent determinations. Additionally, the MBC/MIC ratio was calculated by dividing the MBC value by the respective MIC values of the tested compound against a* S. aureus* isolate.

### 2.5. Determination of Cell Cytotoxicity and Selectivity Index (SI) Values

The cytotoxicity of each active derivative was evaluated using MTT assay as described previously [28]. Three types of cell lines were used in this study; Vero (kidney-like cell line), WRL-68 (liver-like cell line), and 3T3 (skin fibroblast). Briefly, cells were cultured in DMEM and supplemented with 5% foetal bovine serum and 1% penicillin-streptomycin. Growing cells were harvested and seeded in 96-well microplate at the density of 2000 cells/well. Cells were allowed to attach and spread overnight prior to their incubation with the active compounds at various concentrations. After 72 hrs of incubation with the compounds, MTT assay was carried out to determine the number of viable cells relative to the control. Paclitaxel, an established cytotoxic anticancer drug, was used as the positive control. Each experiment was conducted in triplicate with three independent experiments. 50% inhibition concentration (IC_50_) values were determined from the corresponding dose response curve. Selectivity index (SI) values for the active compound were also determined by dividing the IC_50_ value by MIC value.

## 3. Results and Discussion

**Figure 1:**
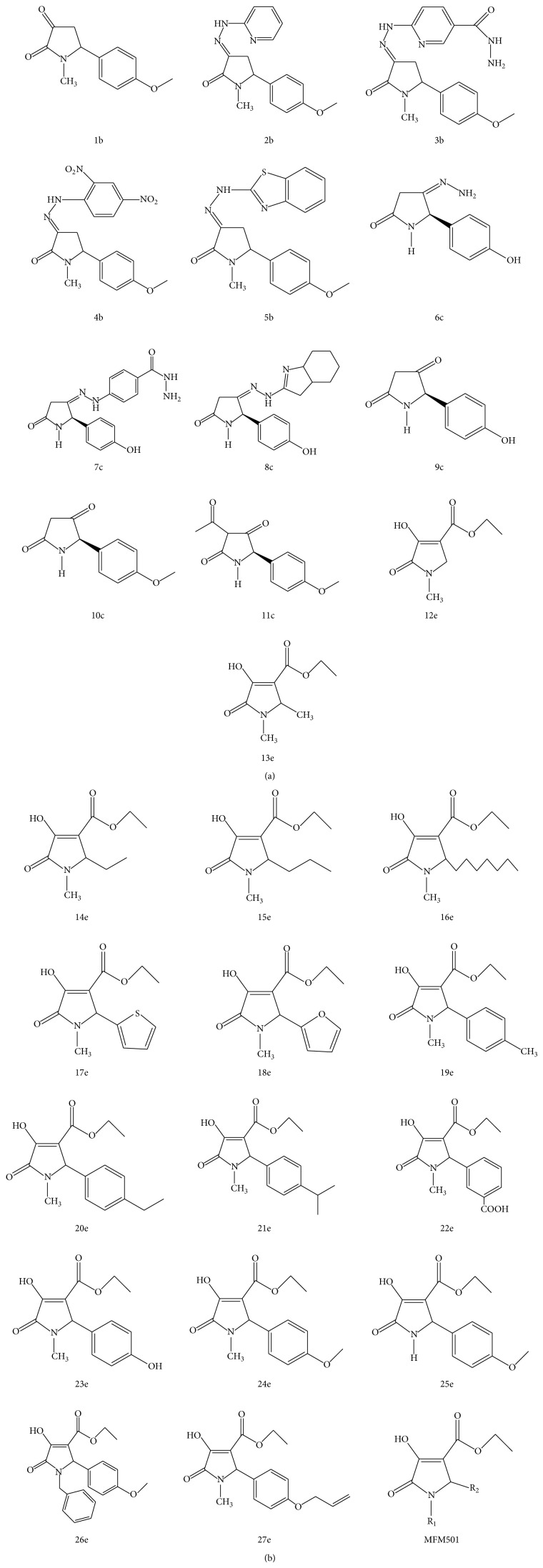
List of the 28 synthetically produced codonopsinine derivatives used in this study.

**Figure 2:**
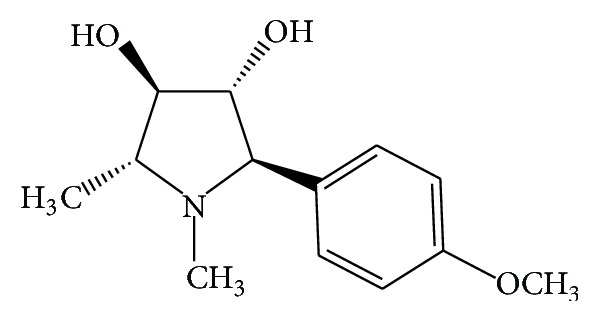
Chemical structure of codonopsinine.

In present investigation, 28 derivatives of codonopsinine analogues were synthesized via Mannich one-pot reaction or Lacey-Dieckmann intramolecular cyclization approach as reported before [22, 23]. The structures of all the 28 analogs were depicted in Figure 1. For initial structure-activity relationship (SAR) comparison studies of the naturally derived codonopsinine (Figure 2), some chemical modifications on the respected derivatives were performed which include installation of different substitution on the aromatic ring (except compounds 12e–16e). In addition, some different hydrazone analogs (2b–8c) were also successfully synthesized and evaluated.

Based on the preliminary evaluation in Table 1, only MFM 501 showed a good inhibitory activity with MIC value of 31.3 *μ*g/mL against all four test isolates. This is followed by compounds 16e and 26e with MIC values between 125 and 250 *μ*g/mL. On the other hand, compound 19e displayed a poor inhibitory activity with MIC values of 500 to 1000 *μ*g/mL. No inhibitory activity was detected from the other pyrrolidine compounds even at concentrations as high as 1000 *μ*g/mL. Based on these results, MFM 501 was selected to be evaluated further using additional* S. aureus* isolates. Nevertheless, as seen in Table 1, vancomycin is still the best choice of drug against the tested MRSA/MSSA isolates.

In Table 2, MFM 501 showed good inhibitory activity with MIC value of 15.6 *μ*g/mL against eight MRSA isolates and one MSSA isolate. Following that, a higher MIC value of 31.3 *μ*g/mL against 35 MRSA and 11 MSSA isolates was observed. On the other hand, UM6, a MSSA isolate, displayed a less potent inhibition with MIC value of 62.5 *μ*g/mL while another two MRSA isolates, D2 and D5, gave higher MIC values of 125 *μ*g/mL towards MFM 501. Nevertheless, MFM 501 did display a weak inhibitory activity towards a MRSA reference strain, ATCC 700699, with MIC value of 250 *μ*g/mL. This particular strain (ATCC 700699) has been described to exert intermediate resistance against vancomycin [29] while BAA-1556 is a CA-MRSA strain that causes more than 98% of skin and soft tissue infection in the United States [30]. The MIC results suggest that MFM 501 has inhibition ability against both CA- and hospital-acquired- (HA-) MRSA but is less effective against vancomycin-intermediate-resistant* S. aureus* (VISA).

An expert review has suggested that extracts with MIC value of >1000 *μ*g/mL have little clinical relevance since a number of relatively inert substances may display antibacterial activity at such a high concentration [9]. Additionally, compounds that exhibited MIC < 64 *μ*g/mL are considered active and could be used for a direct comparison of activities between compound classes [9] although a more recent review by the same author suggests that only compounds displaying MIC values < 10 *μ*g/mL might be considered of interest to the pharmaceutical industries [31].

For the MBC evaluation, 36 MRSA and nine MSSA isolates exhibited a high MBC value of 500 *μ*g/mL while nine MRSA and four MSSA isolates showed a lower MBC value of 250 *μ*g/mL against MFM 501. In addition, only one MRSA isolate, D5, displayed the lowest MBC value of 125 *μ*g/mL. Most of the isolates exhibited an eight- to 32-fold increment of MIC to MBC values against MFM 501. Overall, MFM 501 exhibited a good inhibitory activity with MIC values of 15.6 to 31.3 *μ*g/mL against 55* S. aureus* isolates (43 MRSA and 12 MSSA isolates) while displaying MBC values between 250 and 500 *μ*g/mL against 58* S. aureus* isolates (45 MRSA and 13 MSSA isolates) out of the 59 isolates used in this study. From the 59* S. aureus* isolates tested, MFM 501 exhibited MBC/MIC ratio of 8 and 16 against 49 isolates and MBC/MIC ratio of 32 against six isolates. MFM 501 also displayed MBC/MIC ratio of two on a couple of MRSA isolates (D2 and ATCC 700699) and MBC/MIC ratio of one against a single MRSA isolate (D5).

Previous studies have suggested that an antimicrobial agent is considered bacteriostatic when the MBC/MIC ratio is ≥4 while being bactericidal when the MBC/MIC ratio is ≤4 [32–34]. Additionally, an isolate which showed MBC/MIC ratio of ≥32 is considered tolerant or resistant to the used anti-infective agent [33, 35]. However, the same studies also mentioned that numerous technical problems and other factors could affect the determination of MBC/MIC ratio such as the growth rate of the microbe, antimicrobial penetration, pH level, and temperature [32, 33]. Based on findings of the mentioned studies, MFM 501 may possess a bacteriostatic mechanism of action against MDR MRSA and MSSA isolates. However, resistance against MFM 501 from MRSA could develop since six MRSA isolates out of 46 MRSA (13%) showed MBC/MIC ratio of 32.

As seen in Table 3, MFM 501 did not exhibit any significant cytotoxicity against all three normal cell lines (Vero, WRL-68, and 3T3) with IC_50_ value of >625 *μ*g/mL after 72 hrs of incubation. Following that, a SI value of 19.97 was obtained by dividing the IC_50_ value by MIC value of 31.3 *μ*g/mL against all three normal cell lines. Additionally, a higher SI value of 40.1 could be obtained if a MIC of 15.6 *μ*g/mL was applied. Nevertheless, a much lower SI value would be observed if MIC values of 62.5 *μ*g/mL, 125 *μ*g/mL, and 250 *μ*g/mL from four isolates (UM6, D2, D5, and ATCC 700699) were applied to give out SI values of 10, 5, and 2.5, respectively. Previous studies have recommended that pure compounds should have a SI value of >10 to have relevant biological efficacy and to be considered significant for further testing [36–38].

Previous anti-MRSA studies have shown lower MIC values but with higher cytotoxicity values against similar cell lines, as compared to MFM 501. For example, Moreira Osório et al. [39] described four synthetic compounds that exhibited MIC values of 15.6–125 *μ*g/mL with IC_50_ values of 7.81–125 *μ*g/mL against Vero cell line. Following that, another study reported two pyrimidinedione derivatives that displayed MIC value of two and 16 *μ*g/mL but with IC_50_ of 52 and 42 *μ*g/mL against 3T3 cell line, respectively [40]. Lastly, five alkylresorcinol compounds from* Merulius incarnatus* showed MIC values between five and 13 *μ*g/mL but both compounds exhibited IC_50_ value of 25 *μ*g/mL against Vero cell line [41]. In brief, although MFM 501 has a higher MIC value of 15.6–31.3 *μ*g/mL, it has the advantage of no evident cytotoxicity activity even at >625 *μ*g/mL which could lead to possible oral anti-MRSA application.

Another advantage of using pyrrolidine compounds as potential anti-MRSA agent is that most of the studies only involve modifications of the pyrrolidine functional group on the chemical structures of well-known wide-spectrum antibiotics such as fluoroquinolones and carbapenems [42–45] rather than modification on the pyrrolidine structure itself as reported previously [46]. It was suggested by several authors that the pyrrolidine ring plays an important role for potent antibacterial activity and high penicillin-binding-protein (PBP) affinity in the targeted microbes used in their studies [42, 43, 47, 48]. Furthermore, MFM 501 has a good yield of 20 to 60% as exemplified by the other similar pyrrolidine compounds previously reported using a one-pot reaction [22]. Besides giving out good yield of intended compounds, the advantages of one-pot reaction schemes include reduction of the time required to set up the reactions, removal of the need to isolate unstable intermediates, and reduction of the time required for purifications which would lead to lowering the cost of overall reaction and lessening wastage to the environment [49, 50].

## 4. Conclusion

Our data demonstrated that synthetically derived intermediate compounds based on natural products may play an important role in the discovery of new anti-infective agents against MRSA. Furthermore, a one-pot reaction to produce MFM 501 would suggest that mass production is economically possible and would lead to a time- and cost-effective technique. Nevertheless, further pharmacodynamic and* in vivo* toxicology studies are needed to ascertain their potential therapeutic applications.

## Figures and Tables

**Table 1:** Inhibitory activity of codonopsinine derivatives against a panel of *S. aureus* isolates.

Compounds	MIC (*μ*g/mL)			
	N441	U949	ATCC 25923	ATCC 33591
1b–5b	>1000	>1000	>1000	>1000
6c–11c	>1000	>1000	>1000	>1000
12e–15e	>1000	>1000	>1000	>1000
16e	125	125	250	125
17e-18e	>1000	>1000	>1000	>1000
19e	500	500	1000	500
20e–25e	>1000	>1000	>1000	>1000
26e	125	250	250	125
27e	>1000	>1000	>1000	>1000
MFM 501	31.3	31.3	31.3	31.3
Oxacillin	500	500	0.24	250
Vancomycin	1.56	1.56	0.78	3.13

**Table 2:** MIC, MBC, and MBC/MIC values for MFM 501 against additional *S. aureus* isolates.

MRSA isolates	MIC (*µ*g/mL)	MBC (*µ*g/mL)	MBC/MIC ratio
N391, N829, N850, U949, UM3, UM9, UM10	31.3	500	16
UM13, HN1, UM3, UM10, UM13, UM14, HN3	31.3	500	16
HN5, HN6, HN12, A1, HS770, HS3175, HS3178	31.3	500	16
A2, A3, A4, A7, C1, C4, C8, ATCC 33591	31.3	500	16
HN4, HN7, HN8, A8, C5, D3	31.3	250	8
N441, N1406, HN13, A9, C9, D1	15.6	500	32
HN14, BAA-1556	15.6	250	16
D5	125	125	1
D2	125	250	2
ATCC 700699	250	500	2

MSSA isolates	MIC (*µ*g/mL)	MBC (*µ*g/mL)	MBC/MIC ratio

ATCC 25923, UM9, HN9, HN10, HN11	31.3	500	16
A5, A6, B6	31.3	500	16
HN8, B1, C6	31.3	250	8
ATCC 35556	15.6	250	16
UM6	62.5	500	8

**Table 3:** Cytotoxicity and SI values of MFM 501 against three normal cell lines.

Cell lines	IC_50_	SI (IC_50_/MIC^*^)
Vero	>625	19.97
WRL-68	>625	19.97
3T3	>625	19.97

^*^MIC values of 31.3 *µ*g/mL were chosen to calculate the selectivity index (SI) value.
